# Fuelling the Fight from the Gut: Short-Chain Fatty Acids and Dexamethasone Synergise to Suppress Gastric Cancer Cells

**DOI:** 10.3390/cancers17152486

**Published:** 2025-07-28

**Authors:** Radwa A. Eladwy, Mohamed Fares, Dennis Chang, Muhammad A. Alsherbiny, Chun-Guang Li, Deep Jyoti Bhuyan

**Affiliations:** 1 NICM Health Research Institute, Western Sydney University, Penrith, NSW 2751, Australia; d.chang@westernsydney.edu.au (D.C.); c.li@westernsydney.edu.au (C.-G.L.); 2 Department of Pharmacology, Faculty of Pharmacy, Egyptian Russian University, Badr City 11829, Egypt; 3 Sydney Pharmacy School, The University of Sydney, Sydney, NSW 2006, Australia; mohamed.metwaly@sydney.edu.au; 4 Pharmaceutical Chemistry Department, Faculty of Pharmacy, Egyptian Russian University, Badr City 11829, Egypt; 5 Pharmacognosy Department, Faculty of Pharmacy, Cairo University, Cairo 11562, Egypt; 6 Freedman Foundation Metabolomics Facility, Innovation Centre, Victor Chang Cardiac Research Institute, Darlinghurst, NSW 2010, Australia; 7 School of Science, Western Sydney University, Penrith, NSW 2751, Australia

**Keywords:** gastric cancer, gastric adenocarcinoma, postbiotics, gut microbiome, dexamethasone, apoptosis, proteomics

## Abstract

**Background:** Short-chain fatty acids (SCFAs), microbial metabolites also known as postbiotics, are essential for maintaining gut health. However, their antiproliferative effects on gastric cancer cells and potential interactions with conventional therapies remain underexplored. This study aimed to investigate the effects of three SCFA salts—magnesium acetate (A), sodium propionate (P), and sodium butyrate (B)—individually and in combination (APB), as well as in combination with dexamethasone (Dex), on AGS gastric adenocarcinoma cells. **Methods:** AGS cells were treated with PB, AP, AB, APB, Dex, and APB+Dex. Cell viability was assessed to determine antiproliferative effects, and the IC_50_ of APB was calculated. Flow cytometry was used to evaluate apoptosis and necrosis. Reactive oxygen species (ROS) levels were measured to assess oxidative stress. Proteomic analysis via LC-MS was performed to identify differential protein expression and related pathways impacted by the treatments. **Results:** SCFA salts showed significant antiproliferative effects on AGS cells, with APB exhibiting a combined IC_50_ of 568.33 μg/mL. The APB+Dex combination demonstrated strong synergy (combination index = 0.76) and significantly enhanced growth inhibition. Both APB and APB+Dex induced substantial apoptosis (*p* < 0.0001) with minimal necrosis. APB alone significantly increased ROS levels (*p* < 0.0001), while Dex moderated this effect in the combination group APB+Dex (*p* < 0.0001). Notably, the APB+Dex treatment synergistically targeted multiple tumour-promoting mechanisms, including the impairment of redox homeostasis through SLC7A11 suppression, and inhibition of the haemostasis, platelet activation network and NF-κB signalling pathway via downregulation of NFKB1 (−1.34), exemplified by increased expression of SERPINE1 (1.99) within the “Response to elevated platelet cytosolic Ca^2+^” pathway. **Conclusions:** These findings showed a multifaceted anticancer mechanism by APB+Dex that may collectively impair cell proliferation, survival signalling, immune modulation, and tumour microenvironment support in gastric cancer.

## Simple Summary

Finding better ways to treat gastric cancer remains a significant challenge due to limited treatment options and drug resistance. In this study, we investigated whether naturally occurring short-chain fatty acids (SCFAs), produced by the gut microbiota from food, could enhance the efficacy of the standard drug dexamethasone against gastric cancer. Using gastric cancer cells, we found that the combined treatment was more effective at stopping cancer cell growth, promoting cell death, and disrupting tumour survival pathways. This suggests that pairing common gut microbiota-derived metabolites such as SCFAs with conventional drugs may offer new hope for more effective gastric cancer therapies in the future.

## 1. Introduction

Cancer continues to be one of the most significant global health challenges, with gastric cancer ranking as the fifth most common malignancy and the third leading cause of cancer-related mortality worldwide [1,2]. Multiple risk factors contribute to gastric cancer, including chronic *Helicobacter pylori* infection, dietary habits high in salt-preserved foods, smoking, excessive alcohol intake, and genetic predispositions [1]. Beyond its physical burden, gastric cancer can cause profound psychological distress, with many patients experiencing anxiety, depression, and significant impacts on their quality of life [3]. Despite significant advancements in treatment options such as chemotherapy, radiotherapy, and immunotherapy, the prognosis for patients, particularly those with advanced or metastatic gastric cancer, remains poor [4]. Conventional therapies often come with severe side effects, suboptimal effectiveness, and the eventual development of drug resistance, highlighting the urgent need for more effective and less toxic therapeutic strategies [5,6]. Recent advances in cancer therapy have increasingly focused on harnessing the potential of postbiotics derived from gut microbial communities, particularly short-chain fatty acids (SCFAs). These metabolites, including acetate, propionate, and butyrate, are produced during the fermentation of dietary fibre and have demonstrated anti-inflammatory, immunomodulatory, and anticancer properties [7,8,9]. The majority of SCFAs are produced in the large intestine through bacterial fermentation and are subsequently absorbed into the bloodstream [10,11]. Total SCFA concentrations in the intestine, primarily the colon, typically range from 20 to 140 mM, with higher concentrations in the proximal colon (70–140 mM) and lower concentrations in the distal colon (20–70 mM) [10,11]. In gastric cancer, SCFAs have shown potential in modulating tumour cell metabolism, enhancing apoptosis, and inhibiting proliferation [12]. This makes SCFAs attractive candidates for combination therapies aimed at improving the efficacy of conventional cancer treatments while mitigating their side effects.

Sodium butyrate (B) has emerged as a functionally effective anticancer agent due to its ability to induce apoptosis, arrest cell cycles, and alter gene expression by inhibiting histone deacetylases (HDACs) at physiologically relevant concentrations [13]. Research has shown that B can sensitise cancer cells to chemotherapeutic drugs such as 5-Fluorouracil (5-FU), oxaliplatin, and cisplatin, increasing apoptosis while decreasing drug resistance in colorectal, cervical, and gastric cancers [13,14]. For example, combining B and cisplatin in gastric cancer has been shown to enhance apoptosis and reduce cell migration and invasion [14]. Sodium propionate (P), although less studied than B, has also demonstrated promising anticancer effects, particularly in enhancing apoptosis through modulation of critical signalling pathways, such as the Wnt/β-catenin and NF-κB pathways [15,16]. Dexamethasone (Dex), a glucocorticoid commonly used in cancer treatment for its immunomodulatory and anti-inflammatory effects, has been shown to enhance the action of chemotherapeutic agents [17]. However, its use in treating solid tumours such as gastric cancer remains limited. Combining Dex with SCFAs could offer a novel approach by simultaneously targeting cancer cell survival mechanisms, modulating the tumour microenvironment, and enhancing immune responses.

The concept of synergistic interactions between SCFAs and chemotherapeutic agents is gaining traction [14]. Synergy occurs when the combined effect of two or more agents exceeds the sum of their individual effects, leading to enhanced therapeutic outcomes [14]. For instance, the pairing of B with cisplatin enhanced apoptosis and decreased tumour volume in cervical cancer models [14]. Likewise, when combined with 5-FU or oxaliplatin, B has been shown to potentiate the effects of these drugs, improving their efficacy and reducing drug resistance in colorectal cancer [14]. Given the diverse mechanisms by which SCFAs modulate cancer cell behaviour, their combination with conventional therapies like Dex could pave the way for more effective treatments in gastric cancer. To build upon existing research, the current study employs a combination index model to assess synergy between SCFAs and Dex, utilising flow cytometry, reactive oxygen species (ROS) analysis, and proteomics to investigate their molecular interactions. Unlike previous studies that primarily focus on individual SCFAs or their general anticancer properties, this research aims to elucidate their combined effects with Dex, providing a more comprehensive mechanistic understanding. Additionally, our previous findings demonstrated that B exhibited the most potent antiproliferative effects among SCFAs and showed synergy with Dex in AGS gastric adenocarcinoma cells [18]. The current study extends that work by exploring the role of multiple SCFA salts in combination with Dex, analysing their effects on cancer cell metabolism, oxidative stress, and proteomic alterations. These insights may contribute to the development of SCFA-based adjuvant therapies for gastric cancer.

## 2. Materials and Methods

### 2.1. Chemicals and Drug Preparation

All solvents utilised in this study were of analytical grade and sourced from Sigma Aldrich (Castle Hill, NSW, Australia). A, P, B and Dex standards were also obtained from Sigma Aldrich (Castle Hill, NSW, Australia). All reagents used in the proteomics analyses were purchased from Thermo Fisher Scientific (Waltham, MA, USA) unless stated otherwise.

### 2.2. Cell Culture

AGS gastric adenocarcinoma (CRL-1739, ATCC) andHS738.St/Int stomach intestinal (CRL-7869, ATCC) cell lines were obtained from the American Type Culture Collection (ATCC, Manassas, VA, USA). AGS cells were cultured in ATCC-formulated F-12K medium (Kaighn’s Modification of Ham’s F-12) containing 2 mM L-glutamine, 1500 mg/L sodium bicarbonate, and 10% foetal bovine serum (FBS; Bio-Strategy PTY, Campbellfield, VIC, Australia), with the addition of 1% penicillin and streptomycin (Sigma-Aldrich, Castle Hill, NSW, Australia). HS738.St/Int cells were grown in ATCC-formulated DMEM (Dulbecco’s Modified Eagle Medium), which included 4.5 g/L glucose, L-glutamine, sodium pyruvate, and 10% FBS, also supplemented with 1% penicillin and streptomycin. Both cell lines were maintained at 37 °C in a 5% CO_2_ environment, with cell maintenance performed every 48–72 h to allow for the formation of confluent monolayers.

### 2.3. Cell Viability Assays

The viability of AGS cells following treatment with six-point dose responses in 1:2 dilutions starting from 3000 µg/mL of the postbiotics combinations (AP (1:1), AB (1:1), PB (1:1), APB (1:1:1)) and 200 µg/mL Dex were assessed using the Alamar Blue assay, as described in previous studies [19,20]. Briefly, 100 µL of cells were seeded at a density of 10^5^ cells/mL in 96-well plates. After 24 h, the cells were treated with the test compounds and incubated for an additional 72 h. At the end of the incubation, the culture medium was removed, and 100 µL of a 0.1 mg/mL Alamar Blue solution (prepared from a 1 mg/mL resazurin stock in PBS, diluted 1:10 with serum-free media) was added to each well. Fluorescence was measured using a microplate spectrophotometer (BMG CLARIOstar, Mornington, VIC, Australia), with excitation at 555 nm and emission at 595 nm. All compounds were tested in triplicate, with the negative control set to 100% cell viability.

### 2.4. Synergy

Dex was combined with the postbiotics combination APB in 1:1 (3000 μg/mL of APB + 100 μg/mL of Dex) for combination index (CI) analyses. The potential interactions between Dex and APB were analysed using the CI model, and CompuSyn version 2.0 (Biosoft, El Cajon, CA, USA) was used for the CI calculations based on the median-effect equation, derived from the mass action law [20]. In the current study, the combination of APB and Dex was investigated using a six-point dose–response curve with the CI model.

### 2.5. Flow Cytometry Analyses of Apoptotic Profiles

The effect of APB (3000 µg/mL), Dex (200 µg/mL), and APB+Dex (3100 µg/mL), on apoptosis in AGS gastric adenocarcinoma cells was assessed using an annexin V/7-AAD kit (#ab214663, Abcam) as previously described [20,21]. Briefly, AGS cells (1 × 10^6^ cells/10 mL) were cultured in T75 flasks at 37 °C with 5% CO_2_ for 24 h. Cells were treated with APB (3000 µg/mL), Dex (200 µg/mL), and APB+Dex, with serum-containing media as the control. After 24 h, cells were harvested, stained with annexin V-CF Blue and 7-AAD, and analysed using a Novocyte 3000 flow cytometer (ACEA Biosciences, San Diego, CA, USA). Data were processed with NovoExpress software (v1.5.0) and visualised using GraphPad Prism (v9.0). Apoptotic profiles were determined by gating live, early apoptotic, late apoptotic, and necrotic cells based on the fluorescence of annexin V and 7-AAD.

### 2.6. ROS Production Analysis

The effect of APB, Dex and APB+Dex on oxidative stress in the AGS gastric adenocarcinoma cancer cells was evaluated using the H2DCFDA (2′,7′-dichlorofluorescein diacetate) Cellular Reactive Oxygen Species (ROS) Detection Assay Kit (#ab113851; Abcam, Melbourne, VIC, Australia) [19]. In brief, AGS cells (2.5 × 10^5^ cells/mL) were seeded in a 96-well plate, allowed to adhere overnight, and then treated with 20 μM H2DCFDA for 45 min to assess ROS levels. After the dye solution was removed, the cells were washed with 1× buffer. The cells were then exposed to 750, 1500, and 3000 µg/mL of APB; 50, 100, and 200 µg/mL of Dex; 3000:100 μg/mL, 1500:50 μg/mL, and 750:25 μg/mL of APB+Dex; and 250 μM of tert-butyl hydroperoxide (TBHP) and incubated at 37 °C for 4 h. The fluorescence was measured immediately at an excitation/emission of 485/535 nm using a microplate spectrophotometer (BMG CLARIOstar, Mornington, VIC, Australia). The fold increase in ROS production was calculated relative to the negative control (cells treated with buffer as per the manufacturer’s instructions).

### 2.7. Liquid Chromatography-Mass Spectrometry, Label Free Quantification Bottom-Up Proteomics Analysis

#### 2.7.1. Cell Culture, Treatment, and Protein Extraction

The AGS adenocarcinoma cells were initially placed in T75 flasks at a concentration of 1.0 × 10^6^ cells/mL and were allowed to incubate overnight at 37 °C in the presence of 5% CO_2_. After removing the media, fresh F-12K medium supplemented with 10% FBS was added, and the cultured flasks were treated with specific doses of APB (3000 µg/mL), Dex (200 µg/mL), and APB+Dex (3100 µg/mL). Treatments were performed in triplicate and incubated for 24 h at 37 °C in a humidified atmosphere with 5% CO_2_. Following incubation, each flask of cells was subjected to a 0.25% *w*/*v* trypsin treatment for 3 min at 37 °C, and the cell culture medium was collected. To neutralise the trypsin, an equal volume of media containing F-12K medium (containing 10% FBS) was added before mixing with the previously collected media. The cells were then spun in a centrifuge at 500× *g* for 5 min at RT. The cell pellets were subsequently washed twice with ice-cold PBS and spun again at 500× *g* for 5 min.

#### 2.7.2. Sample Preparation

The AGS gastric adenocarcinoma cells treated with APB, Dex and APB+Dex were homogenised in 300 µL lysis buffer, sodium deoxycholate (4% *w*/*v*), 50 mM ammonium bicarbonate, pH 7.6) and sonicated using a Sonifiers SFX tip probe (Branson, Danbury, CT, USA) for eight cycles (30 s on and 30 s off) at a frequency of 30% whilst on ice. The debris was removed through centrifugation (Eppendorf, Hamburg, Germany) at 9000× *g* for 5 min, the resulting supernatant was aliquoted, and 4× volume of ice-cold acetone was added to denature and precipitate proteins. Samples were incubated at −30 °C overnight before centrifugation at 10,000× *g* for 20 min, the supernatant was removed, and the protein pellets were air-dried for 3 h. Protein pellets were reconstituted with 100 µL of denaturing buffer (6 M urea, 2 M thiourea, 100 mM HEPES buffer, pH 7.5) and then reduced with dithiothreitol (10 mM final concentration, room temperature, 1 h incubation) and alkylated with iodoacetamide (25 mM final concentration, room temperature, 30 min incubation in darkness) before being diluted 6-fold (50 mM ammonium bicarbonate, pH 7.6). Protein quantification was determined using a Qubit 4 (Invitrogen, Carlsbad, CA, USA) according to the manufacturer’s instructions. Samples were aliquoted to a final concentration of 2 µg, and trypsin (4 µL, 12 ng/µL, Promega, Madison, WI, USA) was added to each sample. Digestion occurred at 37 °C and samples were incubated overnight with shaking at 600 rpm. After digestion, samples were then acidified to 0.1% TFA, concentrated and desalted using C18 Zip-Tips (Millipore, Bedford, MA, USA) per the manufacturer’s instructions. The solvent was removed from the desalted peptides through vacuum using a Concentrator plus (Eppendorf, Hamburg, Germany), and samples were then resuspended in 20 µL of loading buffer (3% (*v*/*v*), acetonitrile, 0.1% (*v*/*v*) formic acid), briefly sonicated and centrifuged at 16,000× *g* for 10 min. Samples were run in triplicates.

#### 2.7.3. Liquid Chromatography-Mass Spectrometry Data Independent Analysis

Samples were separated by nano-LC using an Ultimate 3000 HPLC and autosampler system (Dionex, Amsterdam, The Netherlands) coupled to an in-house built fritless nano 75 μm × 45 cm column packed with ReproSil Pur 120 Å, 1.9 μm, C18 stationary phase (Dr Maisch GmbH, Ammerbuch, Germany). LC mobile phase buffers comprised buffer A: 0.1% (*v*/*v*) formic acid in HPLC-grade water and buffer B: 80% (*v*/*v*) acetonitrile, 0.1% (*v*/*v*) formic acid in HPLC water. Peptides were eluted using a linear gradient from 5% buffer B to 40% buffer B over 60 min, and then an increase to 60% buffer B over 5 min and then, 98% buffer B over 3 min before an isocratic wash at 98% buffer B for 7 min at a flow rate of 300 nL/min. The LC was coupled to a Q Exactive HF-X Orbitrap mass spectrometer (Thermo Fisher Scientific, Waltham, MA, USA). The column voltage was set to 2400 V, the heated capillary set to 300 °C, and no sheath and auxiliary gas flow. Positive ions were generated by electrospray, and the Orbitrap operated in data-independent acquisition mode. A survey scan of 350–1650 *m*/*z* was kept constant, acquired with a resolution at 60,000, and an accumulation target value of 3,000,000 ions, followed by 20 narrow isolations windows covering 350–1650 *m*/*z* with varying widths of 26–589 *m*/*z*, resolution set to 30,000, and an accumulation gain control value of 3,000,000 ions. The stepped normalised higher energy collisional dissociation (HCD) collision energy was set to 22.5, 25 and 27.5%.

#### 2.7.4. Data Processing

The raw data was analysed with SpectronautTM software v. 19.0.240604.62635 (Biognosys, Schlieren, Switzerland) using the directDIATM analysis and were searched against the 2023 Human FASTA file and the Human Gene Annotation file downloaded from the UniProt website (https://www.uniprot.org (accessed on 20 November 2024), 20,595 protein sequence entries and 19,756 gene sequence entries). The search library parameters were the default parameters of the BGS Factory Setting for the directDIATM analysis process; this included the following variable modifications: enzymes/cleavage rules: trypsin, peptide length 7–52, missed cleavages: 2, max variable modifications: 5, fixed modifications: carbamidomethyl (C), variable modifications: acetyl (protein N-term), oxidation (M), PSM FDR: 0.01, peptide FDR: 0.01, protein group FDR: 0.01, directDIA workflow: directDIA+ (deep), all tolerance parameters (ion trap, orbitrap, ToF) calibration search: dynamic, MS1 correction factor: 1, MS2 correction factor: 1. XIC extraction: dynamic, MZ extraction strategy: maximum intensity, single hit definition: by stripped sequence, precursor filtering: identified (Qvalue), quantity type: area, differential abundance testing: unpaired *t*-tests.

### 2.8. Statistical Analysis

Data collection and management were conducted using Microsoft Excel and GraphPad Prism (version 9.0, San Diego, CA, USA) for statistical analysis and visualisation. All data collection and analyses were performed in triplicate, with results presented as the mean ± standard deviation. Statistical significance between the mean values was assessed at *p* < 0.05 using a two-way ANOVA. For multiple comparisons, Tukey and Dunnett’s tests were applied within GraphPad Prism software. Additionally, the IC_50_ value, which indicates the concentration of a drug required to inhibit cell growth by 50%, was determined using nonlinear regression with GraphPad Prism software. The IC_90_ and IC_95_ values were derived from nonlinear regression analysis of the dose–response curves using the CompuSyn version 2.0 (Biosoft, El Cajon, CA, USA). The n = 3 refers to the number of independent biological replicates.

## 3. Results and Discussion

### 3.1. Antiproliferative Activity of Postbiotic Combinations, Standard Immunotherapy and Standard Chemotherapy

In our recent study, we showed that SCFA salts- A, P and B exhibited a significant inhibitory effect (*p* < 0.05) against the AGS gastric adenocarcinoma cells [18]. At 3000 μg/mL, B demonstrated an inhibition value of 100.36 ± 1.23%, followed by P at 95.15 ± 1.99% and A at 81.47 ± 20.28% against AGS cells following a 72 h treatment [18]. Additionally, the combination of Dex and B showed strong synergy at a 2:8 ratio (40 μg/mL Dex + 2400 μg/mL B) with significantly greater inhibitory activity (*p* < 0.05) compared to the mono treatments [18]. Physiologically, acetate, propionate, and butyrate are the three main SCFAs naturally produced together in the human gut through microbial fermentation of dietary fibres [10,11]. Combining all three in this study better reflects these physiological conditions, strengthening the translational relevance of our findings. This multi-SCFA approach may also reveal complementary or additive anticancer mechanisms not fully captured by using butyrate alone.

In the current study, the effect of different combinations of A, P and B was investigated for their antiproliferative activity against AGS cells. Furthermore, the combination of APB+Dex was studied against the AGS gastric adenocarcinoma cells. Table 1 presents the cell growth inhibition (%) of AGS cells when treated with various combinations of the SCFA salts (AP, AB, PB, APB), Dex, and APB+Dex following a 72-treatment using the Alamar Blue assay. This study is among the first to investigate the antiproliferative effects of the combined A and P on AGS gastric adenocarcinoma cells. The AP combination showed modest growth inhibition at higher concentrations (93.75 μg/mL–3000 μg/mL), with the highest inhibition of 59.79 ± 4.32% at 3000 μg/mL. The IC_50_ for AP was found to be relatively high at 1141.13 ± 362.00 μg/mL, indicating weaker potency. The AB combination showed 99.49 ± 0.85% inhibition at 3000 μg/mL and a potent IC_50_ of 446.53 ± 19.55 μg/mL, suggesting a strong anti-proliferative activity against the AGS cells. The PB combination displayed high cell inhibition, reaching 99.22 ± 1.42% at 3000 μg/mL, with an IC_50_ of 421.23 ± 15.31 μg/mL, making it comparable to AB (*p* < 0.05). The APB combination displayed inhibitory action similar to AB and PB (*p* < 0.05), with 95.65 ± 7.90% inhibition at 3000 μg/mL and an IC_50_ of 568.33 ± 82.56 μg/mL.

The cell viability of Hs 738.St/Int normal intestinal cells remained relatively high across all tested concentrations for all three combinations (AP, AB, and PB). At the highest concentration (3000 μg/mL), the viability was 82.39% (AP), 61.08% (AB), and 58.86% (PB). As the concentration decreased, cell viability generally increased, with the highest viability observed at 46.875 μg/mL for AP (175.62%), AB (149.29%), and PB (138.92%), compared to the negative control. These values suggest that the normal cells might have recovered or even proliferated slightly at lower concentrations in response to treatment. This is likely because SCFAs, particularly butyrate, are known to serve as a primary energy source for intestinal epithelial cells, promoting their growth and maintenance of barrier function [22]. This cell viability study suggested that the SCFA, even at their highest tested concentration of 3000 μg/mL, showed less than 50% toxicity against the normal Hs 738.St/Int cells, indicating their therapeutic potential against gastric adenocarcinoma.

SCFAs have been investigated in many studies for their potential to enhance the effectiveness of chemotherapy, radiotherapy, and immunotherapy [23,24]. Previous studies have shown that B, when combined with cisplatin or Dex, can induce apoptosis in various tumour cells, including gastric and cervical cancers, both in vitro and in vivo [18,23]. Here, we sought to study the synergistic potential of the SCFAs (APB) and Dex. We previously reported Dex with potent inhibition of AGS cells, achieving 80.46% inhibition at 200 μg/mL in a dose-dependent manner, with an IC_50_ value of 86.60 ± 11.85 μg/mL [18]. In the current study, the APB+Dex combination (3000 μg/mL of APB + 100 μg/mL of Dex) showed 103.17 ± 3.06% inhibition against AGS cells, with an IC_50_ of 643.30 ± 58.26 μg/mL (Table 2).

The combination of APB and Dex was also tested on normal Hs 738.St/Int human cells, revealing a dose-dependent effect on cell viability. At the highest concentration of APB+Dex (3100 μg/mL), cell viability significantly decreased to 68.57 ± 11.74%, indicating cytotoxicity at this dose. However, at lower concentrations (1550 and 775 μg/mL), cell viability increased to 85.91 ± 10.59% and 110.88 ± 14.35%, respectively, suggesting recovery of cellular activity. Interestingly, at lower concentrations (387.5, 193.75, and 96.875 μg/mL), the combination appeared to stimulate cell proliferation, with viability reaching 115.29 ± 16.81%, 134.46 ± 24.72%, and 159.21 ± 14.16%, respectively, compared to the negative control. Notably, our previous findings demonstrated that B and Dex, when tested individually, exhibited a favourable safety profile, as they did not significantly reduce the viability of normal intestinal cells [18]. B even promoted cell proliferation, likely due to its role as a primary energy source for intestinal epithelial cells. In contrast, the APB+DEX combination exhibited cytotoxic effects at higher concentrations and proliferation at lower doses in the Hs 738.St/Int cells. This suggested that while B and Dex were well tolerated in normal cells, combining APB with Dex altered the cellular response. This may be attributed to mechanisms such as nutrient utilisation or stress-induced compensatory responses in normal intestinal cells [10]. Even at the highest tested concentration of 3100 μg/mL, the APB+Dex combination showed less than 50% cytotoxicity against the normal Hs 738.St/Int human cells, supporting its favourable safety profile at lower doses (when compared with the normal intestinal cell line used in this study). These results underscored the potential therapeutic utility of APB+DEX while highlighting the importance of dose optimisation to minimise toxicity in healthy tissues.

### 3.2. Synergistic Potential of APB with Dex Against the AGS Gastric Adenocarcinoma Cells

The potential synergistic effects of AP, AB, PB, and APB+Dex on AGS cells were analysed using the CI model, as shown in Table 3. The interactions were categorised into three distinct groups: synergistic effects (CI < 1), additive effects (CI = 1), and antagonistic effects (CI > 1). The AP, AB and BP combinations showed CI values of 3.53, 1.14 and 1.41, respectively, indicating antagonistic interactions. The results indicated that the APB+Dex yielded a CI value of 0.76, signifying a strong synergistic interaction. This was particularly evident at the IC_50_, IC_75_, IC_90_, and IC_95_ levels, where the CI values consistently remained below 1, reinforcing the effectiveness of this combination in inhibiting the growth of AGS cells. Interestingly, our previous study demonstrated a similar strong synergistic effect between B and Dex in AGS cells, with a B+Dex ratio of 2:8 (2400 μg/mL B + 40 μg/mL Dex) yielding CI values < 1 compared to their mono treatments [18]. The B+Dex combination achieved 80.32–103.78% inhibition of AGS cell growth while also improving the viability of normal Hs 738.St/Int intestinal cells, highlighting its favourable safety profile (when compared with the normal intestinal cell line used in this study) [18]. The findings from the current study align with our previous results, further supporting the potential of combining Dex with SCFA-based compounds to enhance antiproliferative efficacy while minimising toxicity.

### 3.3. Flow Cytometric Analyses of Apoptotic Profiles of Mono and Combination Therapies

**Figure 1:**
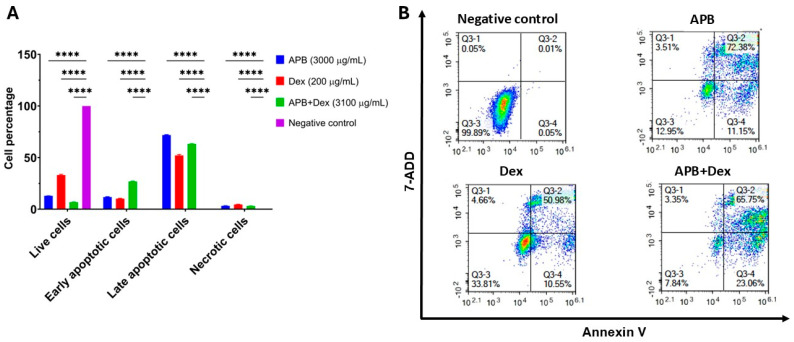
Flow cytometric assessment of the apoptotic profiles of the AGS gastric adenocarcinoma cells after 24 h of treatment. (**A**) The live, early apoptotic, late apoptotic, and necrotic cell percentages after 24 h treatment with APB (3000 µg/mL), APB+Dex (3100 µg/mL), Dex (200 µg/mL), and control (n = 4). (**B**) Represented are the density plots of each drug treatment that is most representative of the average data from the flow cytometric analyses, with Q3-1 = necrotic cells, Q3-2 = late-stage apoptotic cells, Q3-3 = live cells, and Q3-4 = early-stage apoptotic cells. **** indicates *p* < 0.0001 compared to the negative control.

Apoptosis, a type of programmed cell death, plays a crucial role in preventing cancer by eliminating malignant cells from the body [25]. Due to the importance of apoptosis in cancer suppression, many new anticancer therapies aim to target this biological process [25]. Annexin V and 7-AAD were employed to identify apoptosis and necrosis, respectively, using flow cytometry. Annexin V binds to phosphatidylserine, which is exposed on the outer membrane of cells during apoptosis, while 7-AAD, with a high affinity for guanine-cytosine residues, intercalates into double-stranded DNA to indicate necrosis. The effects of the mono treatments APB, Dex, and the combination APB+Dex were investigated using flow cytometric analysis, compared to the negative control (Figure 1). The results are categorised into live cells, early apoptotic cells, late apoptotic cells and necrotic cells.

Dex (200 μg/mL) alone showed a relatively balanced effect, with a moderate proportion of live cells (33.11%), early apoptotic cells (10.17%), and a notable proportion of late apoptotic cells (52.08%). The necrotic cell population remained low (4.64%) in the Dex treatment (*p* < 0.0001), although it was slightly higher than in the combination treatments (Figure 1). Nevertheless, apoptosis was still the dominant cell death mechanism in the Dex-treated cells. APB (3000 μg/mL) treatment resulted in a high proportion of apoptotic cells, including late apoptotic cells (71.91%) and early apoptotic cells (11.81%), indicating a significant induction (*p* < 0.0001) of apoptosis compared to the negative control. The proportion of necrotic cells was low (3.32%), indicating that APB primarily induced apoptosis rather than necrosis, and a small proportion of live cells (12.96%) was observed (Figure 1). Overall, APB induced a substantial amount of apoptosis, predominantly in the late stage, while maintaining a low percentage of necrosis, underscoring its effectiveness in triggering programmed cell death. When combined with Dex, the apoptotic effect was enhanced, particularly in the early stage, and the proportion of live cells decreased, indicating a synergistic interaction between APB and Dex. This APB+Dex combination promoted apoptosis in the AGS gastric adenocarcinoma cells more effectively (*p* < 0.0001) than either agent alone, with minimal necrosis observed. These findings aligned with our previous study on the B+Dex combination, where the synergy between Dex and B also significantly increased apoptosis while keeping necrosis at a low level [18]. Notably, the B+Dex combination (40:2400 μg/mL) resulted in a significant increase in early apoptotic cells (48.97%; *p* < 0.0001) compared to Dex alone, similar to the enhancement of early apoptosis observed in the APB+Dex combination. However, in contrast to the APB+Dex combination, which showed a predominant late apoptotic response, the B+Dex combination favoured early apoptosis. This difference may not necessarily indicate distinct mechanisms of action, but it could suggest that APB+Dex induces apoptosis more rapidly, causing a greater proportion of cells to progress to the late apoptotic stage by the time of measurement.

### 3.4. ROS Production in the AGS Cells After Treatment with Different Concentrations of APB, Dex and APB+Dex

**Figure 2:**
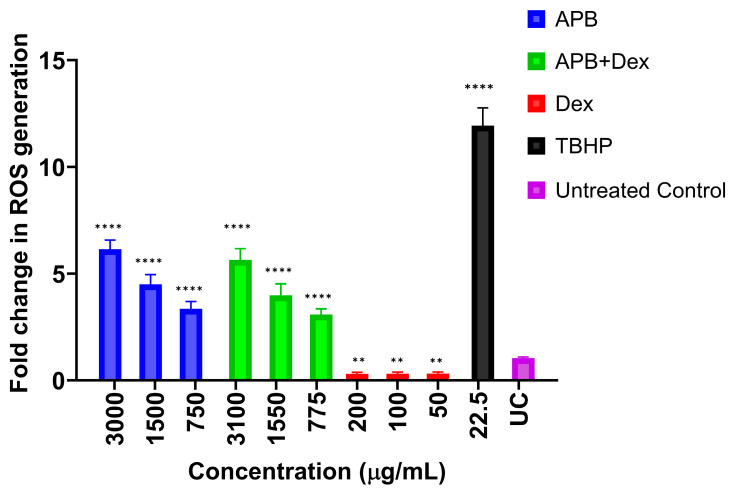
Depicts the fold change in reactive oxygen species (ROS) generation following treatment with various concentrations: 3000 μg/mL, 1500 μg/mL, and 750 μg/mL of APB, and 3000:100 μg/mL, 1500:50 μg/mL, and 750:25 μg/mL of APB:Dex and, 200 μg/mL, 100 μg/mL, and 50 μg/mL of Dex. Additionally, tert-Butyl hydroperoxide (TBHP) (22.5 μg/mL or 250 μM) is included for comparative purposes. The values are expressed as mean ± SD. ** indicates value of *p* ≤ 0.01, **** indicates *p* < 0.0001 compared to the negative control.

Oxidative stress, marked by elevated levels of ROS, is a critical factor in the development and progression of cancer [26]. Therefore, enhancing or reducing ROS levels in cancer cells can be a promising strategy for anticancer therapy [26]. This study evaluated the effects of APB, its combination with Dex, and individual treatments on ROS production in AGS gastric adenocarcinoma cells at different concentrations. TBHP, used as a positive control for ROS induction, resulted in significantly higher ROS levels (11.94-fold) compared to the negative control, which showed baseline ROS levels (0.91). This confirmed the assay’s sensitivity in detecting oxidative stress changes in the cells (Figure 2). At the highest concentration (3000 μg/mL), APB alone significantly increased ROS production (6.14-fold), suggesting a strong pro-oxidative response. When combined with Dex, the ROS levels were moderately reduced to 5.64-fold (*p* < 0.0001), indicating that Dex may mitigate APB-induced oxidative stress. At lower concentrations, a consistent pattern emerged; at 1500 μg/mL, APB alone led to a 4.50-fold increase in ROS, while its combination with APB and Dex (APB+Dex) reduced ROS to 3.98-fold, reinforcing the potential antioxidative role of Dex. The APB+Dex combination at this concentration (1500 μg/mL of APB + 50 μg/mL of Dex) maintained elevated ROS levels at 4.21-fold (Figure 2). At 750 μg/mL, APB alone induced a ROS increase of 3.35-fold, while the combination APB+Dex brought ROS down further to 3.08-fold, suggesting a consistent reduction in oxidative stress by Dex. Lower concentrations of Dex alone (200 μg/mL, 100 μg/mL, 50 μg/mL) consistently showed very low ROS production (0.30–0.31), indicating its strong antioxidative properties (Figure 2).

These findings align with our previous study, which similarly demonstrated that Dex significantly reduced ROS production in AGS cells at all tested concentrations (*p* ≤ 0.01), indicating its potent antioxidative effects [18]. In contrast, B exhibited strong pro-oxidative properties, significantly increasing ROS levels compared to the negative control (*p* < 0.0001). Notably, the Dex+B combination exhibited a distinct ROS modulation pattern, with ROS levels significantly higher than those of Dex alone but lower than those of B alone. This suggested that Dex counteracted some of the oxidative stress induced by B, a pattern that parallels the current study’s findings with APB and Dex.

While B and APB both demonstrated pro-oxidative effects, APB (*p* < 0.0001) induced a stronger ROS response at high concentrations (3000 μg/mL). Dex exhibited a protective antioxidative impact in both studies, reducing ROS when combined with pro-oxidative agents. This consistent ROS-modulating effect of Dex across different SCFA-based combinations suggested its potential role in balancing oxidative stress, which may be crucial for optimising therapeutic efficacy while minimising oxidative damage. Overall, these findings display the importance of ROS regulation in gastric cancer therapy and highlight the potential benefits of combining Dex with pro-oxidative agents to fine-tune oxidative stress responses in AGS gastric cancer cells.

### 3.5. Proteomics Study of the AGS Cells Treated with the Synergistic Combination vs. Mono Treatments

While studies have evaluated the anticancer potential of SCFAs on various cancer cell lines, the molecular mechanisms underlying SCFA combinations remain largely unexplored [27]. In the current study, proteomic analysis was conducted for the SCFA combination APB, Dex, and their combination APB+Dex (Tables S1–S5). The mono and combination treatments were compared to the untreated control to decipher their mechanisms of action against AGS cells as explained in the following sections.

#### 3.5.1. Enrichment Analyses of Differentially Expressed Proteins (DEPs) in APB-Treated AGS Cells Compared to the Untreated Control Cells

**Figure 3:**
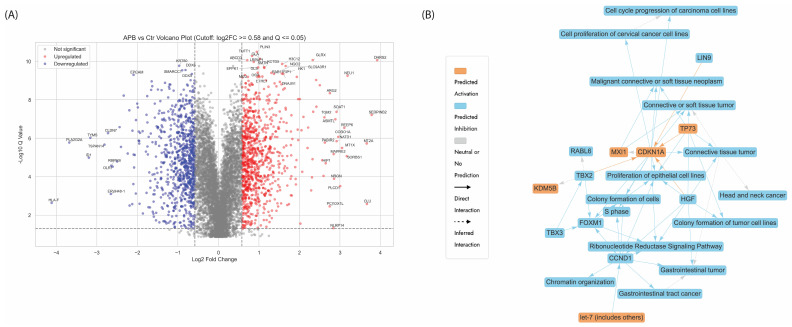
Pathway enrichment analysis using differentially expressed proteins in APB-treated AGS cells compared to control cells. (**A**) Volcano plot showing significantly regulated proteins (Absolute Log_2_FC ≥ 0.58 and Q ≤ 0.05) (**B**) Ingenuity Pathway Analysis (IPA) graphical summary of top enriched and predicted terms.

The volcano plot (Figure 3A) comparing APB-treated AGS gastric cancer cells to untreated control highlights several key proteins with significant differential expression that may underpin APB’s antiproliferative effects. On the upregulated side, CLU (Clusterin), also known as apolipoprotein J, stands out as one of the most highly induced genes (log_2_FC > 3), often associated with stress response, apoptosis, and inhibition of cell growth in certain contexts, including gastric cancer [28]. CLU has been shown to play a protective role in the gastric epithelium by regulating cellular responses to injury and limiting abnormal proliferation during the emergence of spasmolytic polypeptide-expressing metaplasia, a preneoplastic condition [29]. Similarly, SERPINB2 also known as plasminogen activator inhibitor type 2 (PAI-2), and HIP1 (Huntingtin-interacting protein 1-related) are notably upregulated—each implicated in cell death, and or inhibition of metastasis, respectively, refs. [30,31] SERPINB2, has been shown to inhibit tumour metastasis by being present on tumour cell-derived microparticles, thereby reducing the invasive potential of cancer cells [30]. HIP1R functions as a tumour suppressor in gastric cancer by promoting apoptosis and inhibiting the proliferation, migration, and invasion of cancer cells. It achieves this, in part, by modulating the Akt signalling pathway, which is crucial for cell survival and growth [31]. DNAJB1, a heat shock protein, is also elevated and may play a role in proteostasis under therapeutic stress, promoting apoptosis in cancer cells [32].

Conversely, several proteins critical for proliferation and tumour maintenance are downregulated (Figure 3A). Notably, TYMS (Thymidylate Synthase), a key enzyme involved in DNA synthesis and repair, is strongly suppressed [33]. TYMS is frequently overexpressed in various types of cancer and is a well-known contributor to chemotherapy resistance [33]. Its overexpression has been associated with increased genomic instability, a hallmark of cancer progression [34,35]. Therefore, its downregulation in response to APB treatment suggested a potential reduction in genomic instability, contributing to a less favourable environment for tumour growth and survival. EPCAM, involved in cell adhesion and widely recognised as a gastric cancer stem cell marker, is also significantly downregulated, suggesting a loss of proliferative and invasive potential [36]. Other suppressed genes, such as *CLDN7*, *ID1*, and *PLA2G2A*, further reflect diminished epithelial integrity and proliferative signalling, all aligning with anticancer responses [37,38,39].

The graphical summary (Figure 3B) ties multiple dysregulated proteins and pathways to gastric adenocarcinoma biology. Cell cycle regulation, a critical hallmark of cancer, was notably impacted by APB treatment through CCND1 (Cyclin D1) and CDKN1A (p21), both of which are central to the transition from the G1 to the S phase [40,41]. Dysregulation of these proteins is frequently observed in gastric and other gastrointestinal malignancies, where CCND1 is often overexpressed to promote proliferation, while CDKN1A can act as a tumour suppressor or be inactivated [40,41]. In this network, their interplay suggests a mechanistic shift in cell cycle dynamics, directly influencing gastric tumour growth. Figure 3B also highlights tumour-related processes, with connections to terms like gastrointestinal tumour, gastrointestinal tract cancer, and connective tissue tumour, emphasising the relevance to gastric adenocarcinoma. The involvement of proteins such as TP73 and HGF was detected in this study following APB treatment—both known contributors to gastric cancer progression, with TP73 implicated in apoptosis resistance and HGF in invasive growth [42,43]. The proliferation and colony formation cluster, featuring FOXM1, HGF, and RAEL6, underscored increased cellular turnover, a key trait in GIT tumour aggressiveness [43,44]. Additionally, chromatin remodelling components, such as KDM5B and let-7 (miRNA), hint at epigenetic reprogramming often seen in GIT cancers, which affects gene silencing and oncogene activation [45,46]. Lastly, signal transduction pathways, such as the Ribonucleotide Reductase Signalling pathway, are essential for DNA synthesis and repair, processes often hijacked in gastric cancer to sustain rapid cell division [47]. Altogether, the dysregulated genes and pathways in this network mirrored known molecular disruptions in gastrointestinal cancers, suggesting that APB treatment exerted therapeutic effects by restoring balance across these oncogenic circuits.

The canonical pathways (Table S3) displayed chromatin organisation and ribonucleotide reductase signalling are the most significantly dysregulated pathways with a strong negative z-score, indicating suppression. Other inhibited pathways included regulation of endogenous retroelements, and cell cycle checkpoints, suggesting that APB disrupted key proliferative and repair mechanisms, reinforcing its antiproliferative activity.

- Chromatin Organisation

The inhibition of the chromatin organisation pathway following APB treatment represented a pivotal mechanism contributing to its antiproliferative activity in AGS gastric adenocarcinoma cells. Chromatin remodelling is essential for regulating gene expression, DNA repair, and cell cycle progression—all processes frequently dysregulated in cancer [48] (Tables S1, S2 and SI). Among the significantly downregulated genes were the DNA methyltransferase (DNMT3A) and the histone acetyltransferase CREBBP, as well as NSD2 (a histone methyltransferase) (log_2_FC = −1.08, −1.14, and −1.73, respectively), indicating an impaired epigenetic silencing machinery that potentially reverses oncogenic transcriptional programmes [49,50].

Breast Cancer Metastasis Suppressor 1 (BRMS1) is a protein involved in chromatin remodelling and gene expression regulation, particularly in the context of metastasis [51]. It is known to suppress metastasis in various cancers. In gastric cancer, its expression is often reduced; however, overexpression of BRMS1 (log_2_FC = 0.89) (Table S2 and SI) has been shown to inhibit tumour growth and metastasis in vivo [52]. Meanwhile, the suppression of chromatin remodelling complex components, such as SMARCA4, SMARCB1, and PBRM1 (log_2_FC = −0.90, −1.09, and −1.97, respectively), further supported the widespread disruption of nucleosome dynamics (Table S2 and SI) [53,54,55]. Collectively, these alterations indicated a destabilisation of chromatin architecture, resulting in reduced tumour cell proliferation and viability. This chromatin-targeting effect underscored APB’s potential as an epigenetic modulator in gastric cancer therapy.

- Ribonucleotide Reductase Signalling Pathway

APB treatment significantly suppresses the Ribonucleotide Reductase (RNR) signalling pathway, which plays a central role in maintaining the deoxyribonucleotide (dNTP) pool necessary for DNA replication and repair in proliferating and quiescent cells [56]. RNR is composed of the catalytic subunit RRM1 and either the regulatory subunits RRM2 or RRM2B, forming a complex that is tightly regulated during the cell cycle and in response to stress [56]. In cancer, particularly gastric cancer, high RRM2 expression is commonly observed and is associated with enhanced proliferation, angiogenesis, and chemoresistance via pathways such as NF-κB (NFKB1, log_2_FC = −1.34), AKT-mTOR (AKT1S1, log_2_FC = −0.67), and EGFR (log_2_FC = −0.78) (Table S2 and SI) [57,58,59]. In the current study, APB treatment resulted in downregulation of RRM1 (log_2_FC = −1.1), indicating a disruption of nucleotide biosynthesis that likely contributes to cell cycle arrest and impaired tumour DNA synthesis [60]. This inhibition cascaded through related oncogenic pathways. For example, CHEK1, a DNA damage checkpoint kinase that can upregulate RRM2 via E2F1, was also suppressed (log_2_FC = −1.31), reinforcing the downregulation of RNR activity [61]. Similarly, key regulators in RNR expression and function—such as NFKB1 (−1.34), AKT1S1 (–0.67), and CDK6 (log_2_FC = −1.63)—were also downregulated, reflecting a coordinated silencing of survival and proliferative signalling (Table S2 and SI) [57,62,63]. Intriguingly, CDKN1A (p21) was upregulated (log_2_FC = 0.95), suggesting cell cycle arrest at the G1/S checkpoint, which further halts DNA synthesis and aligns with the suppressed RNR activity [41]. Together, these results indicated that APB imposes a multi-level inhibition of dNTP production and cell cycle progression, strongly implicating the RNR pathway as a targetable vulnerability in gastric cancer.

In the broader context of gastric tumour biology, APB-induced downregulation of the SMARCD family members—SMARCD1 (log_2_FC = −0.795), SMARCD2 (−0.677), and SMARCD3 (−1.021)—suggested disrupted chromatin remodelling, a process vital for transcriptional control in cancer cells (Table S2 and SI) [64]. The downregulation of MAPK13 (log_2_FC = −0.863), a mitogen-activated protein kinase implicated in inflammatory and stress responses, further indicated attenuation of signalling pathways that support tumour survival [65]. Additionally, key members of the poly (adenosine diphosphate-ribose) polymerase (PARP) family—PARP9 (−1.017), PARP12 (−1.190), and PARP14 (−1.208)—are significantly suppressed (Table S2 and SI) [66]. While these genes are commonly upregulated in gastric cancer and contribute to tumour progression, stress adaptation, and cell survival, their inhibition may sensitise tumour cells to damage and promote therapeutic response. Notably, PHF10 (−1.773), a component of the chromatin remodelling complex, was also strongly downregulated, reinforcing the global collapse of transcriptional machinery following APB treatment [67].

- Regulation of endogenous retroelements

APB treatment appeared to modulate the regulation of endogenous retroelements, which are typically silenced in normal cells but often become aberrantly activated in cancer through epigenetic dysregulation. Transposable elements—including LINEs (Long Interspersed Nuclear Elements) and SINEs (Short Interspersed Nuclear Elements) can influence oncogenic transcription through enhancer activity or transcription factor binding [68]. In the context of gastric cancer, APB downregulated several proteins associated with retroelement-linked transcriptional regulation. These include CDK6 (log_2_FC = −1.63), a key G1/S checkpoint regulator often influenced by retroelement-driven chromatin changes, and PHF10 (log_2_FC = −1.77), a subunit of the ATP-dependent PBAF chromatin remodelling complex, essential for its interaction with chromatin [63,67]. Additionally, WEE1 (log_2_FC = −1.12), ERBB2 (log_2_FC = −1.07), and EGFR (log_2_FC = −0.78)—each implicated in DNA damage responses and retroelement-associated promoter activation—are suppressed (Table S2 and SI) [69,70]. The anti-apoptotic proteins BIRC5 (log_2_FC = −0.659) and mTOR complex regulator MLST8 (log_2_FC = −0.66) were also downregulated, further supporting reduced cell survival and metabolic activity [71,72]. Finally, the transcription factor subunit NFYC (log_2_FC = −0.61) was suppressed, indicating disrupted regulatory control over retroelement-responsive gene networks (Table S2 and SI) [73].

- Cell Cycle Checkpoint

Disruption of the cell cycle checkpoint pathway is a defining mechanism through which APB exerted its antiproliferative activity in AGS gastric adenocarcinoma cells. Typically, cell cycle checkpoints ensure genomic integrity by coordinating critical transitions such as G1/S and G2/M, with failure in these processes being a hallmark of cancer progression. In the current study, APB treatment led to strong downregulation of multiple key genes involved in checkpoint regulation and mitotic fidelity. These include UBE2C (log_2_FC = −1.75), NSD2 (log_2_FC = −1.73), ZWINT (log_2_FC = −1.63), BRCA1 (BReast CAncer gene 1; log_2_FC = −1.40), and CHEK1 (−1.31)—all of which are typically overexpressed in gastric cancer and promote cell proliferation, chromosomal segregation, and DNA damage repair (Table S2 and SI) [49,74,75,76,77,78,79]. Their suppression reflected a broad collapse in checkpoint surveillance and genome maintenance functions, potentially leading to mitotic catastrophe and apoptosis in rapidly dividing tumour cells. Additional downregulated regulators such as WEE1 (−1.12), PLK1 (−1.07), MAD2L1 (−0.96), CENPE (−0.81), and AURKB (−0.77) further highlighted the inhibition of mitotic entry and spindle checkpoint integrity (Table S2 and SI) [40,69,80,81,82,83]. Collectively, these proteins play essential roles in ensuring proper kinetochore function, centrosome duplication, and chromosome segregation. Notably, many of these factors are normally upregulated in gastric cancer and contribute to tumour aggressiveness, poor prognosis, and therapy resistance.

#### 3.5.2. Enriched Pathways of DEPs in Dex Treated AGS Gastric Adenocarcinoma Cells Compared to Control

**Figure 4:**
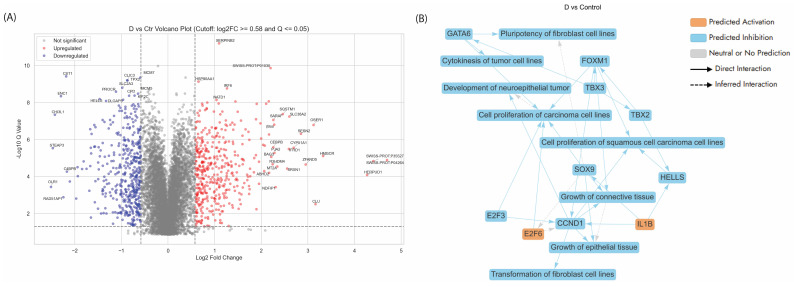
Pathway enrichment analysis using differentially expressed proteins in Dex-treated AGS cells compared to control cells. (**A**) Volcano plot showing significantly regulated proteins (Absolute Log_2_FC ≥ 0.58 and Q ≤ 0.05) (**B**) Graphical summary of top predictions in an Ingenuity Pathway Analysis (IPA) Interpret report presented in the form of a simple network.

Dex, a potent synthetic glucocorticoid, has been shown to significantly affect various pathways and biological processes (Figure 4, Table S4). The volcano plot (Figure 4A) comparing Dex-treated AGS gastric cancer cells to untreated control revealed a distinct pattern of gene expression changes, highlighting Dex’s broad regulatory effects. A substantial number of genes were significantly downregulated (blue), including key cell cycle and DNA repair regulators such as RAD51AP1, MCM5, TPX2, and HELls, indicating suppression of proliferative and genomic maintenance pathways. Conversely, a cluster of genes was significantly upregulated (red), including CLU (Clusterin), HERPUD1, MT2A, CEBPB, and NDFIP1, many of which are linked to stress response, apoptosis, or metabolic regulation. The presence of strongly upregulated chaperones and antioxidant-related genes, along with suppression of proliferation-associated factors, suggested that Dex induces a stress-adaptive, anti-proliferative transcriptional programme in the AGS gastric adenocarcinoma cells.

The graphical summary (Figure 4B) of differentially expressed proteins (DEPs) in AGS cells treated with Dex revealed several key biological processes and regulatory networks disrupted by the treatment. The central themes identified included inhibition of cell proliferation, suppression of tumour-promoting transcriptional networks, and modulation of cytokine-responsive gene expression. Core regulators such as CCND1, E2F3, FOXM1, SOX9, and GATA6 are significantly affected, many of which are commonly overexpressed in gastric cancer and are associated with tumour growth, cell cycle progression, and epithelial transformation [84]. The interplay of these proteins with cytokine mediators (e.g., IL1B) further suggested a strong immunomodulatory component of Dex’s action.

The canonical pathways (Table S4) in AGS gastric cancer cells following Dex treatment highlighted both positively and negatively regulated processes, indicating Dex’s dual impact on metabolic and cell cycle-related signalling. The most significantly upregulated pathways include activation of protein expression by *SREBF* (SREBP, Sterol Regulatory Element-Binding Proteins) and multiple branches of cholesterol biosynthesis, suggesting that Dex strongly enhanced lipid metabolic programmes. Given that cancer cells often rewire lipid metabolism to support membrane synthesis and rapid proliferation, this shift may reflect either a compensatory adaptation or Dex-driven metabolic stress. Conversely, several key proliferative pathways showed strong inhibition, most notably cell cycle checkpoints, activation of the pre-replicative complex, and cell cycle control of chromosomal replication. These pathways are critical for ensuring DNA replication fidelity and progression through S-phase and mitosis—core processes often dysregulated in gastric and other cancers.

- Activation of SREBF-Mediated Cholesterol Biosynthesis in Dex-Treated AGS Cells

Dex treatment in AGS gastric adenocarcinoma cells resulted in a robust activation of the SREBF (SREBP)-mediated transcriptional programme, as evidenced by the significant upregulation of key proteins involved in the cholesterol biosynthesis pathway. SREBPs are master regulators of lipid homeostasis, primarily controlling the expression of enzymes required for cholesterol and fatty acid synthesis [85]. Dex enhanced the nuclear activity of SREBP1/2, driving the expression of canonical downstream targets including HMGCR (log_2_FC = 3.327), CYP51A1 (2.695), SQLE (2.064), FDFT1 (1.889), SC5D (1.737), and MSMO1 (1.761) (Table S2 and SI) [85,86,87]. These genes encoding these proteins span multiple stages of the mevalonate pathway and both branches of cholesterol synthesis—via desmosterol (Bloch pathway) and lathosterol (Kandutsch–Russell pathway)—highlighting widespread pathway engagement.

Increased cholesterol synthesis intermediates could sensitise cells to ferroptosis or oxidative stress, both being anticancer mechanisms [88]. Dex treatment resulted in the downregulation of key SREBP co-factors—including SP1 (log_2_FC = −0.594), CREBBP (−0.645), and NFYB (−0.669)—all of which are typically upregulated in cancer (Table S2 and SI) [89,90]. These co-factors are essential for full transcriptional activation, and their suppression could limit the tumour-promoting effects of the cholesterol biosynthesis programme [91]. Moreover, repression of RXRA (−0.718), a nuclear receptor involved in lipid signalling and inflammatory crosstalk, may further modulate this axis toward a tumour-suppressive phenotype [92]. Altogether, these findings suggested that Dex-induced activation of cholesterol biosynthesis may not reflect metabolic support for tumour growth but rather a form of stress-induced reprogramming. Enhanced cholesterol biosynthesis may reflect a cellular stress response or a mechanism promoting membrane synthesis in proliferative cells.

- Proteins related to the cell cycle

Dex treatment also led to the dysregulation of key proteins involved in apoptosis, DNA repair, cell cycle progression, and autophagy [93]. Among these, Survivin BIRC5 (log_2_FC = −1.36), an inhibitor of apoptosis protein, was downregulated, which aligned with studies indicating that Dex promotes apoptotic pathways by reducing survival signals in cancer cells [71]. Similarly, the suppression of cell division cycle 20 (CDC20; log_2_FC = −1.56) and ubiquitin-conjugating enzyme E2 C (UBE2C; log_2_FC = −1.58)—key players in cell cycle progression—pointed to Dex-induced mitotic arrest, a mechanism often exploited to curb cancer cell proliferation. Upregulation of CDKN1A (log_2_FC = 1.01) and downregulation of polo-like kinase 1 (PLK1; log_2_FC = −0.94) reflected Dex’s dual role in promoting cell cycle arrest and limiting mitotic progression (Table S2 and SI) [94,95,96]. CDKN1A, also known as p21, is a cyclin-dependent kinase inhibitor that regulates the G_1_/S checkpoint, preventing cells from progressing through the cell cycle. This effect, coupled with PLK1 suppression, a key regulator of mitotic entry, suggested that Dex enforced a halt in cell proliferation of AGS cells in our study. The p21 was reported to be a negative regulator of p53 stability [41]. Furthermore, the downregulation of BRCA1 (log_2_FC = −1.93) and TP53 (log_2_FC = −0.75), critical mediators of the DNA damage response—indicated a potential attenuation of repair mechanisms, which might sensitise cancer cells to chemotherapy or further DNA damage. Notably, BRCA1 was also downregulated by APB treatment (log_2_FC = −1.40). The significant upregulation of P62, known as SQSTM1 (log_2_FC = 2.46), correlated with its role as a mediator in autophagy and cellular stress pathways (Table S2 and SI) [97]. This could indicate an adaptive response of AGS cells to Dex-induced stress, possibly balancing autophagy with cell death mechanisms. Additionally, the marked upregulation of P35527 (log_2_FC = 4.41) and downregulation of C4BPB (log_2_FC = −2.17) and CD320 (log_2_FC = −2.11) further emphasised the impact of Dex on the cellular microenvironment, particularly influencing immune-modulatory and nutrient uptake pathways (Table S2 and SI) [98,99].

- Ferroptosis

Ferroptosis is a form of programmed cell death distinct from apoptosis, necrosis, or autophagy, characterised by the accumulation of lipid peroxidation products and lethal ROS derived from iron-dependent reactions [100]. This process is tightly regulated and plays a significant role in various biological contexts, including cancer, neurodegeneration, and ischemia–reperfusion injuries [100]. The upregulation of HMGCR (log_2_FC = 3.32), HMOX1 (log_2_FC = 0.83), ACSL4 (log_2_FC = 0.74), FTH1 (log_2_FC = −0.75), GCLC (log_2_FC = 0.85), GCLM (log_2_FC = 0.87), TXNRD1 (log_2_FC = 0.98) in combination with the downregulation of SLC11A2 (log_2_FC = −0.65), TFRC (log_2_FC = −0.64), and OLR1 (log_2_FC = −2.51) indicated induction of ferroptosis in the AGS cells upon Dex treatment (Table S2 and SI) [101]. This observation aligned with previous investigations conducted by us and others [18,102].

#### 3.5.3. Enriched Pathways Using DEPs of APB+Dex Combination Treated AGS Cells vs. Mono Treatments

Based on the promising antiproliferative and apoptotic effects of the APB+Dex combination against AGS gastric adenocarcinoma cells, we analysed the proteins linked to these activities. We compared the proteomic profiles of combination therapy with those of the individual treatments and the untreated control.

**Figure 5:**
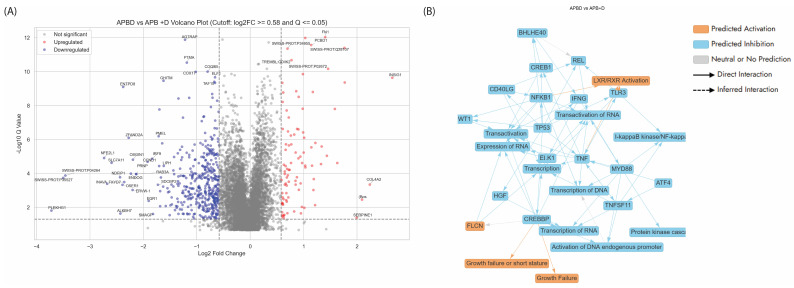
Pathway enrichment analysis using differentially expressed proteins in APB+Dex treated AGS compared to single treatments. (**A**) Volcano plot showing significantly regulated proteins (Absolute Log_2_FC ≥ 0.58 and Q ≤ 0.05). (**B**) Graphical summary of top predictions in an Ingenuity Pathway Analysis (IPA) Interpret report presented in the form of a simple network.

The volcano plot comparing APB+Dex versus APB and Dex mono treatments alone highlighted key DEPs that reveal the molecular effects of adding dexamethasone to APB therapy in AGS gastric cancer cells (Figure 5A). Several proteins, including INSIG1, PCBD1, and COL4A2, were significantly upregulated (log_2_FC > 0.58, Q ≤ 0.05), suggesting enhanced extracellular matrix organisation, cholesterol homeostasis, and metabolic regulation, possibly linked to Dex’s modulatory effects [103,104,105]. Conversely, a large number of proteins involved in cell proliferation, oxidative stress, and immune response—such as AGTRAP, COX17, TAF15, ELF1, NFE2L1, OSGIN1, and CCND1—were significantly downregulated, indicating suppression of proliferative and inflammatory pathways upon combination treatment [40,106,107,108,109,110]. The marked repression of CCND1, a critical cell cycle regulator, and EGR1, an early growth response gene, supported a Dex-mediated antiproliferative shift [111]. Overall, this comparison demonstrated that Dex addition to APB intensified the suppression of tumour-promoting pathways while modestly activating stress-adaptive and metabolic programmes, underscoring its potential to enhance therapeutic efficacy. A summary of other dysregulated proteins upon the combination treatment is listed in Table 4.

The graphical summary of differentially expressed proteins in the APB+Dex versus APB-only and Dex-only treatment highlighted significant transcriptional reprogramming and immune modulation driven by the addition of Dex (Figure 5B, Table S5). Central to this network are key regulators, such as CREBBP, TP53, NFKB1, and MYD88, which coordinate critical processes, including transcriptional activation, immune signalling, and inflammation [112,113,114,115]. Downregulation of transcription factors (ELK1, REL, ATF4) and transcriptional co-activators, such as CREBBP, suggested a global reduction in RNA and DNA transcription, potentially curbing tumour-promoting gene expression [116]. Notably, suppression of the TNF-NFKB1-MYD88 axis implied attenuation of the I-kappaB kinase/NF-kappaB cascade, a pathway often hyperactivated in gastric and other cancers to promote survival and inflammation [117]. Moreover, the network reflected a decrease in pro-inflammatory cytokine signalling through downregulation of IFNG, TLR3, and related mediators, consistent with Dex’s immunosuppressive effects [118]. This may contribute to the observed suppression of transcription and transactivation processes across multiple genes. Additionally, the reduced activity of growth and proliferation-associated proteins (HGF, FLCN, CD40LG) aligns with predictions of impaired cell growth and development, as visualised in Figure 5B by links to phenotypes such as “Growth Failure” and “Short Stature” [119].

**Table 4:** List of the dysregulated proteins in APB+Dex-treated AGS gastric adenocarcinoma cells.

Gene	Protein	Log_2_FC	Role	Ref
***OSER1***	Oxidative stress-responsive serine-rich protein 1	−2.39	Noted for its role in the negative regulation of intracellular signal transduction.	[120]
***DKK1***	Dickkopf-related protein 1	−1.54	Overexpressed in cancer, affecting Wnt signalling pathways. Overexpression correlates with poor survival in gastric cancer.	[121]
***FOXO1***	Forkhead box protein O1	−1.21	A tumour suppressor transcription factor linked to cancer.	[122]
***EPHA4***	Ephrin type-A receptor 4	−1.07	A receptor tyrosine kinase promoting cancer progression.	[123]
***ABL1*** ***ABL2***	Tyrosine kinase ABL1 and ABL2	−0.79 −0.76	Proto-oncogenes involved in cell differentiation, division, and adhesion and is linked various cancers, especially leukaemia. Altered signalling associated with gastric cancer.	[124]
***MET***	Hepatocyte growth factor receptor	−0.78	A proto-oncogene involved in several cancers, including gastric cancer. Overexpression and mutations linked to poor prognosis in gastric cancer.	[125]
***SRC***	Proto-oncogene tyrosine-protein kinase Src	−0.68	Associated with numerous cancers through oncogenic signalling. Promotes gastric cancer progression through activation of oncogenic pathways.	[126]
***STAT1***	Signal transducer and activator of transcription 1-alpha/beta	0.85	Influences cancer progression and immune responses	[127]
***DUSP10***	Dual specificity protein phosphatase 10	1.33	Regulates pathways connected to cancer development.	[128]
***CDKN2D***	Cyclin-dependent kinase 4 inhibitor D	1.18	A cyclin-dependent kinase inhibitor linked to multiple cancers.	[129]
***EPCAM***	Epithelial cell adhesion molecule	−1.04	Cell adhesion and signalling; upregulated in gastric tumours for proliferation and metastasis.	[36]
***GNAQ***	Guanine nucleotide-binding protein G(q) subunit alpha	−0.64	Oncogene in G-protein signalling; implicated in tumour progression.	[130]
***GNAS***	Guanine nucleotide-binding protein G(s) subunit alpha isoforms XLas	−0.73	Oncogenic signalling driver; mutated in some gastric cancers.	[130]
***MAFG***	Transcription factor MafG	−0.65	Transcription factor in oxidative stress response; linked to oncogenesis.	[131]
***TSPAN1, TSPAN6, TSPAN8, TSPAN14, TSPAN15, TSPAN31***	Tetraspanin	−0.78 to −1.49	Roles in cell signalling, adhesion, and metastasis.	[132]

- Proteins Related to Homeostasis and Tumour Microenvironment Regulation

APB+Dex treatment in AGS gastric cancer cells significantly altered the expression of proteins involved in haemostasis, extracellular matrix remodelling, and oxidative stress regulation, as reflected in the top canonical pathways enriched in the analysis. Most notably, the “Response to elevated platelet cytosolic Ca^2+^” and “LXR/RXR Activation” pathways—ranked as the most significantly upregulated—underscored a coordinated activation of calcium and lipid-mediated signalling that impacts coagulation, immune regulation, and vascular interaction. These processes are central to tumour progression and metastasis but may also act as targets for tumour suppression when tightly modulated.

The upregulation of key coagulation and fibrinolysis-related proteins—*SERPINE1* (log_2_FC = 1.993), *F5* (1.765), *F13A1* (1.63), *FGA*, *FGB*, *FGG* (1.458, 1.338, 1.172), and *FN1* (1.404)—pointed to enhanced clotting and extracellular matrix (ECM) stabilisation (Table S2 and SI) [133]. These changes, tied to “Formation of Fibrin Clot (Clotting Cascade)” and “Cell surface interactions at the vascular wall”, suggested a shift toward a more adhesive and structurally reinforced microenvironment that may hinder tumour cell dissemination. In parallel, key tumour-supportive proteins were notably suppressed upon APB+Dex treatment. SLC7A11 (log_2_FC = −2.60) is a cystine/glutamate antiporter that plays a dual role: it supports antioxidant defence by importing cystine, which is essential for glutathione (GSH) synthesis and thus helps limit oxidative stress by neutralising reactive oxygen species (ROS) [134,135]. Suppression of SLC7A11 lowers intracellular GSH levels, reduces GPX4 activity, and thereby promotes lipid peroxidation—a hallmark of ferroptotic cell death. SLC7A11 was strongly downregulated, suggesting APB+Dex disrupted redox homeostasis, potentially sensitising cancer cells to oxidative damage [134,135]. Similarly, FSTL3 (log_2_FC = −1.62), a glycoprotein implicated in regulating the extracellular matrix and associated with metastasis in gastric cancer, was also reduced. Its repression may contribute to decreased tumour cell proliferation and invasion by limiting cell–matrix interactions [136,137]. Together, these haemostasis- and matrix-related changes illustrated how APB+Dex may reprogram the tumour microenvironment, impair survival mechanisms, and reinforce cytotoxic and anti-metastatic effects in gastric cancer.

- Proteins related to Transcription Regulation and Cell Growth Modulation

Pleckstrin homology domain-containing family G member 2 (PLEKHG2) is a key regulator of Rho GTPases, which are pivotal in cytoskeletal remodelling, cell migration, and intracellular signal transduction. These functions are critical for maintaining cellular architecture and responding to environmental stimuli [138]. PLEKHG2 was reported to be upregulated in gastric cancer [139]. The downregulation of PLEKHG1 (log_2_FC = −3.72) suggested that APB+Dex is effective in targeting pathways critical to gastric cancer progression. By disrupting PLEKHG1-mediated functions, APB+Dex might induce structural and signalling deficits, contributing to its antiproliferative activity. APB+Dex combination treatment also resulted in the downregulation of Tensin-4 (log_2_FC = −1.35) (Table S2 and SI). Tensin-4 (TNS4, also known as Cten) is an oncogene involved in regulating cell adhesion, migration, and signalling pathways and has been associated with cancer progression [140]. Interestingly, we previously reported that mono-treatment with B resulted in the upregulation of both TNS4 and PLEKHM1 [18]. The downregulation of structural and signalling proteins such as PLEKHG1 (log_2_FC = −3.72) and TNS4 (log_2_FC = −1.35) following APB+Dex treatment in AGS gastric adenocarcinoma cells underscored a broader transcriptional repression of oncogenic regulators linked to cytoskeletal dynamics, adhesion, and migration (Table S2 and SI).

Insulin Induced Gene 1 (*INSIG1*) is a key regulator of intracellular signalling, particularly in sterol biosynthesis and metabolic homeostasis [103]. It modulates the activity of sterol regulatory element-binding proteins (SREBPs), which are critical for lipid metabolism [103]. A previous study suggested that reduced expression of the *INSIG1* gene may be involved in the development or progression of gastric cancer [141]. In AGS cells treated with APB+Dex, the upregulation of INSIG1 (log_2_FC = 2.66) and SRBP1 (log_2_FC = 1.19) could indicate a reversal of this cancer-associated silencing (Table S2 and SI) [141]. Given its potential tumour-suppressive role, this restoration may indicate a reactivation of normal cellular regulatory processes, contributing to an antiproliferative effect [141]. A list of dysregulated genes by the combination treatment is illustrated in Table 4 and Figure 5.

- Proteins related to amino acid transport across the plasma membrane

Solute carrier (SLC) proteins are membrane transport proteins that facilitate the movement of nutrients, ions, drugs, and neurotransmitters across cell membranes [142]. APB+Dex treatment significantly downregulated key amino acid transporters across the plasma membrane, disrupting essential metabolic pathways in gastric cancer cells. Among these, SLC7A11 showed the most pronounced decrease (Log_2_FC = −2.60), impacting its crucial role in cystine/glutamate exchange and redox balance [135]. This transporter is typically upregulated in gastric cancer to combat oxidative stress and prevent ferroptosis, underscoring its role in tumour survival [135]. Similarly, the downregulation of SLC7A1 (Log_2_FC = −1.26) indicated impaired uptake of cationic amino acids, such as arginine, which is essential for cancer cell proliferation and survival [135].

The neutral amino acid transporters SLC1A4 (Log_2_FC = −1.20), SLC7A5 (Log_2_FC = −0.71), and SLC43A2 (Log_2_FC = −1.20) were also significantly suppressed after APB+Dex treatment. SLC1A4 and SLC43A2 facilitate neutral amino acid uptake, essential for metabolic adaptations in proliferative cancer cells, while SLC7A5 supports the import of large neutral amino acids such as leucine, fuelling tumour growth and is associated with poor prognosis in gastric cancer (Table S2 and SI) [142]. Together, these changes reflected a comprehensive disruption of amino acid transport and metabolic reprogramming critical for tumour growth and oxidative stress resistance. The coordinated suppression of these transporters highlighted their potential as therapeutic targets in gastric cancer treatment, especially under metabolic stress induced by APB+Dex therapy. 

**Figure 6:**
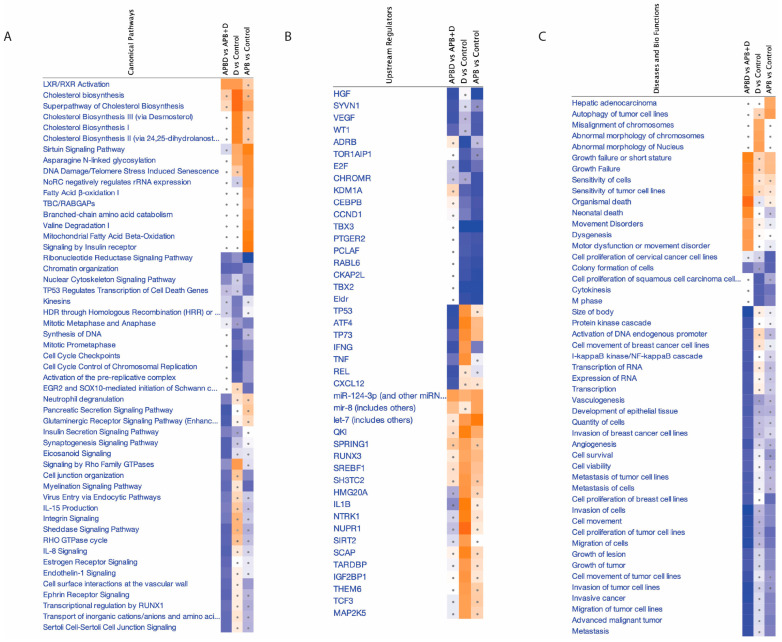
Comparative pathway analysis of APB, Dex, and their combination (APB+Dex) in AGS gastric cancer cells using Ingenuity Pathway Analysis (IPA). Heatmaps show (**A**) affected canonical pathways, (**B**) predicted upstream regulators, and (**C**) disease and bio-function impacts. Orange indicates predicted activation, and blue indicates predicted inhibition (Q ≤ 0.001, |z-score| ≥ 2.5). Some pathways are trimmed as being too long to be listed in this figure which could be retrieved in the Supplementary File (Tables S3–S5).

The disease association analysis for APB+Dex treatment (Figure 6C) in AGS gastric cancer cells highlighted a strong inverse correlation with gene expression signatures typically found in a variety of solid tumours, particularly hepatocellular carcinoma, non-haematological solid tumours, and extracranial or non-melanoma solid tumours, as evidenced by prominent negative z-scores (blue bars). These findings suggested that APB+Dex potentially downregulated gene programmes that are characteristically upregulated in these cancers, including gastric malignancies. Since gastric cancer shares oncogenic pathways and molecular hallmarks with these solid tumour types, such as dysregulated proliferation, angiogenesis, and extracellular matrix remodelling, the observed transcriptional reversal strongly supported the potential antitumor efficacy of APB+Dex in gastric adenocarcinoma.

Figure 6 provides a comparative pathway overview of the effects of APB, Dex, and their combination (APB+Dex) on AGS gastric adenocarcinoma cells, analysed using Ingenuity Pathway Analysis (IPA). Panel A shows that the combination treatment significantly modulated multiple canonical pathways that are crucial for tumour growth and survival. Notably, pathways related to chromatin organisation, cell cycle checkpoints, DNA damage repair, and ribonucleotide reductase signalling were more strongly inhibited by APB+Dex than by either monotherapy, indicating a coordinated suppression of cancer cell proliferation and genome maintenance. The combination treatment shows stronger predicted inhibition of key oncogenic drivers such as HGF, TP53, TNF, IFNG, and VEGF compared to APB or Dex alone (Panel B). This suggested that combining SCFAs with Dex may more effectively disrupt multiple signalling axes that promote tumour growth, inflammation, immune evasion, and angiogenesis.

Panel C focuses on disease and biological functions. It demonstrated that the combination treatment exerts a stronger predicted inhibitory effect on critical cancer-related processes, including tumour metastasis, migration, invasion, angiogenesis, and overall tumour growth, compared to monotherapies. These collective results implied that APB+Dex achieved a multi-targeted anticancer response, enhancing the suppression of pathways and regulators that were only partially affected by APB or Dex alone.

Together, this pathway analysis supported the conclusion that integrating multiple SCFAs with Dex not only amplifies cytotoxic effects but also broadens the scope of disrupted oncogenic mechanisms. This provided a stronger rationale for exploring SCFA-based combination strategies as complementary interventions alongside conventional therapies in gastric cancer.

## 4. Conclusions, Limitations and Future Directions

In conclusion, this study demonstrated the potent antiproliferative effects of the SCFA salts acetate (A), propionate (P), and butyrate (B), both individually and in combination with Dex, in AGS gastric adenocarcinoma cells. Among the individual SCFAs, B showed the most substantial growth-inhibitory effect, while the combined treatment (APB) with Dex exhibited pronounced synergistic activity, significantly enhancing antiproliferative effects. This strong synergy suggested that APB combinations could serve as promising adjuncts in gastric cancer therapy, potentially improving treatment outcomes by increasing drug efficacy while lowering required dosages and minimising resultant side effects. Flow cytometry confirmed that these effects were primarily due to apoptosis, with minimal necrosis, indicating a targeted mode of cell death induction. APB treatment also elevated intracellular ROS levels, with Dex partially modulating this increase when used in combination. Proteomic profiling by LC-MS provided insights into the underlying molecular mechanisms, identifying key proteins involved in apoptosis, autophagy, and immune regulation. Notably, the APB+Dex combination targeted critical pathways related to cell cycle regulation and redox homeostasis, potentially sensitising gastric cancer cells to oxidative stress and ferroptosis.

While Dex is a clinically approved corticosteroid with well-established bioavailability and widespread use in cancer therapy, achieving comparable SCFA concentrations systemically through oral or intravenous routes remains challenging due to their rapid metabolism and clearance. Physiologically, SCFAs naturally reach millimolar levels locally in the colon via microbial fermentation; however, systemic plasma levels are significantly lower. Therefore, for clinical translation, alternative strategies—such as targeted delivery, encapsulation, or local administration—may be necessary to achieve therapeutic concentrations at tumour sites while minimising systemic exposure. Beyond pharmacological use, the current study highlighted the potential of dietary and gut microbiota-based strategies to naturally increase endogenous SCFA levels, which could synergise with conventional therapies such as Dex to support gastric cancer treatment. However, more studies are essential to define dietary interventions and gut microbiota-based approaches that naturally increase SCFA levels in the gut. A limitation of this study was the use of an immortalised cell line (Hs 738.St/Int) as a model for normal intestinal epithelium, which may not fully replicate the behaviour of primary human intestinal cells. Nevertheless, it provided valuable initial insight into the selectivity and safety of SCFA-based combinations toward normal intestinal cells. Overall, these findings supported the potential of APB+Dex combinations as an effective adjunct strategy for gastric cancer treatment. Further investigations using appropriate in vivo models and patient-derived organoids are needed to validate these promising results and advance their clinical translation.

## Figures and Tables

**Table 1:** Cell growth inhibition (%) against the AGS gastric adenocarcinoma and cell viability (%) of the Hs 738.St/Int normal intestine cell lines at different concentrations of AP combination (magnesium acetate A: sodium propionate P), AB (magnesium acetate A: sodium butyrate B), PB (sodium propionate P: sodium butyrate B), for 72 h using the Alamar Blue assay (n = 3).

Conc. μg/mL 1:1	Cell Growth Inhibition (%) of AGS Cells			Cell Viability (%) of HS738.St/Int		
	PB	AP	AB	PB	AP	AB
1500 + 1500	99.22 ± 1.42 ^a^_w_	59.79 ± 4.32 ^a^_x_	99.49 ± 0.85 ^a^_y_	58.86 ± 9.52 ^a^_x_	82.39 ± 12.30 ^a^_x_	61.08 ± 13.45 ^a^_z_
750 + 750	91.14 ± 1.78 ^a^_w_	37.67 ± 9.68 ^b^_x_	90.08 ± 1.86 ^a^_x_	78.04 ± 4.98 ^a^_w_	93.48 ± 12.89 ^a^_x_	84.38 ± 13.01 ^a^_x_
375 + 375	75.93 ± 1.92 ^b^_w_	29.31 ± 6.50 ^b^_x_	76.62 ± 2.81 ^b^_y_	81.09 ± 10.51 ^a^_w_	107.71 ± 33.81 ^a^_y_	102.51 ± 17.25 ^a^_y_
187.5 + 187.5	51.36 ± 7.50 ^c^_w_	23.87 ± 6.76 ^b^_w_	49.95 ± 7.31 ^c^_x_	100.62 ± 16.88 ^a^_w_	143.38 ± 26.78 ^a^_x_	103.77 ± 10.35 ^a^_x_
93.75 + 93.75	25.11 ± 8.21 ^d^_w_	19.20 ± 7.46 ^b^_x_	24.28 ± 7.26 ^d^_y_	127.45 ± 26.75 ^a^_x_	149.37 ± 9.05 ^a^_y_	122.20 ± 16.51 ^a^_z_
46.875 + 46.875	9.79 ± 7.25 ^e^_w_	11.98 ± 12.23 ^b^_x_	12.01 ± 8.06 ^e^_y_	138.92 ± 9.91 ^a^_x_	175.62 ± 4.32 ^a^_y_	149.29 ± 21.90 ^a^_z_
**IC_50_**	**421.23 ± 15.31**	**1141.13 ± 362.00**	**446.53 ± 19.55**	**NA**	**NA**	**NA**

“NA” indicates the IC_50_ value could not be calculated as the highest tested concentration did not inhibit the normal HS738.St/Int cells by 50%. ^a,b,c,d,e^ The different superscript values in the same column for each cell line indicate a statistically significant difference (*p* < 0.05) compared to the highest concentration (3000 μg/mL) within the same treatment group. _w,x,y,z_ The different subscript values in the same row for each cell line indicate a statistically significant difference (*p* < 0.05) between the treatment groups.

**Table 2:** Cell growth inhibition (%) against AGS gastric adenocarcinoma at different concentrations of the APB combination, Dex, and the APB+Dex combination for 72 h using the Alamar Blue assay (n = 3). The table also presents the cell viability (%) of Hs 738.St/Int normal intestinal cell lines for the APB+Dex combination.

Conc. μg/mL	Cell Growth Inhibition (%) of AGS Cells	Conc. μg/mL	Cell Growth Inhibition (%) of AGS Cells	Conc. μg/mL APB + Dex	Cell Growth Inhibition (%) of AGS Cells	Cell Viability (%) of HS738.St/Int
	APB		Dex		APB + Dex	
3000	95.65 ± 7.90 ^a^_x_	200	80.46 ± 8.08 ^a^_x_	3000 + 100	103.17 ± 3.06 ^a^_x_	68.57 ± 11.74 ^a^_x_
1500	86.25 ± 8.42 ^a^_x_	100	39.38 ± 24.51 ^b^_x_	1500 + 50	87.81 ± 5.57 ^b^_x_	85.91 ± 10.59 ^a^_x_
750	65.54 ± 4.91 ^b^_x_	50	-	750 + 25	62.42 ± 5.34 ^c^_x_	110.88 ± 14.35 ^a^_y_
375	40.28 ± 8.05 ^c^_x_	25	-	375+ 12.5	43.49 ± 10.93 ^d^_x_	115.29 ± 16.81 ^a^_y_
187.5	22.20 ± 8.55 ^d^_x_	12.5	-	187.5 + 6.25	28.70 ± 11.91 ^e^_x_	134.46 ± 24.72 ^a^_y_
93.75	19.20 ± 10.38 ^d^_x_	6.25	-	93.75 + 3.13	16.46 ± 9.71 ^f^_x_	159.21 ± 14.16 ^a^_y_
**IC_50_**	**568.33 ± 82.56**	**IC_50_**	**86.60 ± 11.85**	**IC_50_**	**643.30 ± 58.26**	**NA**

“NA” indicates the IC_50_ value could not be calculated as the highest tested concentration did not inhibit the HS738.St/Int cells by 50%. ^a,b,c,d,e,f^ The different superscript values in the same column for each cell line indicate a statistically significant difference (*p* < 0.05) compared to the highest concentration (3000 μg/mL). _x,y_ The different superscript values in the same row for the AGS gastric adenocarcinoma cell line indicate a statistically significant difference (*p* < 0.05) between the treatment groups.

**Table 3:** Drug interaction analysis of Dex and APB combinations in AGS gastric adenocarcinoma cells. The bold numbers (CI values < 1) indicate synergistic interactions between Dex and APB.

Combination Index (CI) Values at				
Combinations	IC_50_	IC_75_	IC_90_	IC_95_
AP 1:1 (1500 μg/mL A + 1500 μg/mL P)	3.53	7.28	17.13	31.66
AB 1:1 (1500 μg/mL A + 1500 μg/mL B)	1.14	1.37	1.80	2.19
BP 1:1 (1500 μg/mL B + 1500 μg/mL P)	1.41	1.68	2.11	2.51
APB + Dex (3000 μg/mL APB + 100 μg/mL Dex)	**0.76**	**0.48**	**0.31**	**0.23**

IC = inhibitory concentration.

## Data Availability

The raw and processed data have been deposited to the ProteomeXchange Consortium via the PRoteomics IDEntifications (PRIDE) repository with the dataset identifier PXD061822.

## Supplementary Materials

The following supporting information can be downloaded at https://www.mdpi.com/article/10.3390/cancers17152486/s1.
