# Supplementary material for: Fuelling the Fight from the Gut: Short-Chain Fatty Acids and Dexamethasone Synergise to Suppress Gastric Cancer Cells
Source: Cancers (Basel). 2025 Jul 28;17(15):2486. doi: 10.3390/cancers17152486 (PMC12346683; doi:10.3390/cancers17152486)

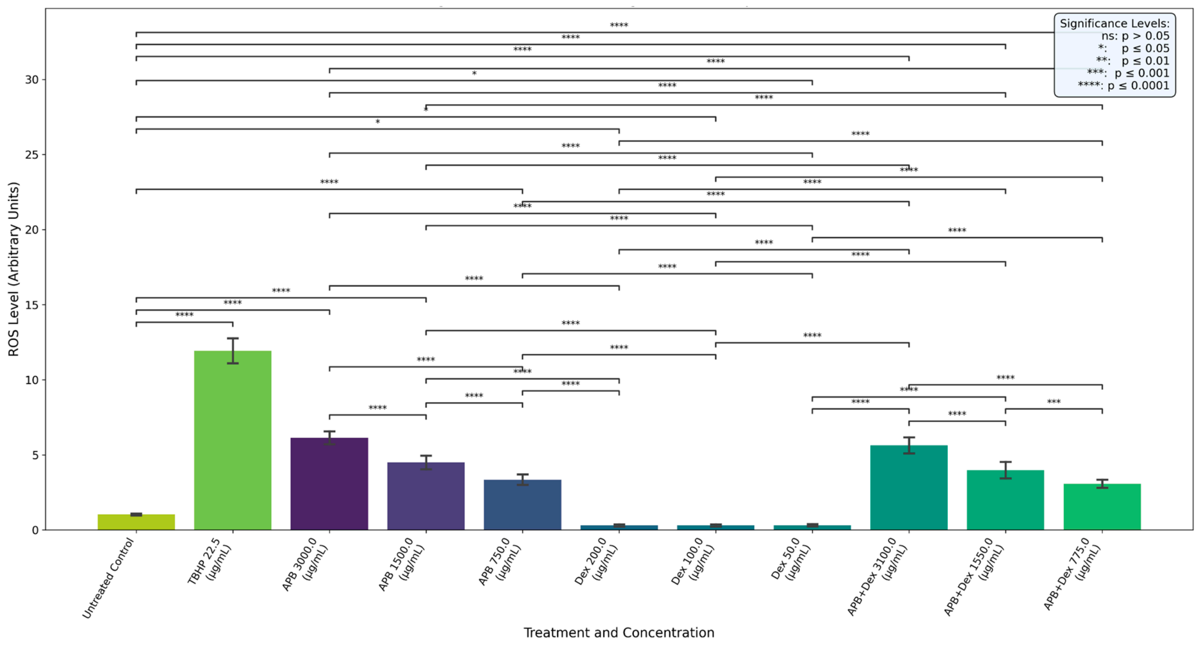

Figure S1. Depicts the fold change in reactive oxygen species (ROS) generation following treatment with various concentrations: 3000 µg/mL, 1500 µg/mL, and 750 µg/mL of APB, and 3000:100 µg/mL, 1500:50 µg/mL, and 750:25 µg/mL of APB:Dex and, 200 µg/mL, 100 µg/mL, and 50 µg/mL of Dex. Additionally, tert-Butyl hydroperoxide (TBHP) (22.5 µg/mL or 250 µM) is included for comparative purposes. The values are expressed as mean ± SD.

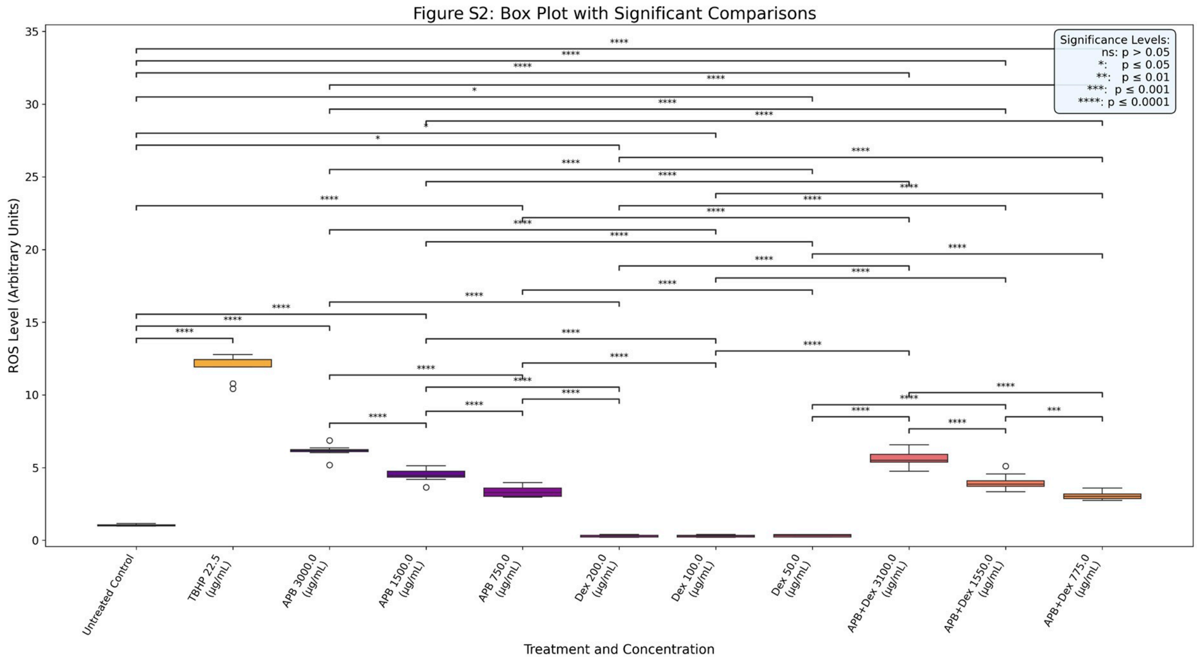

Supplement: Supplementary file 1 [file cancers-17-02486-s001.zip › cancers-3742662-supplementary figures.pdf]
